# Supplementary material for: Modeling glioblastoma heterogeneity as a dynamic network of cell states
Source: Mol Syst Biol. 2021 Sep 16;17(9):e10105. doi: 10.15252/msb.202010105 (PMC8444284; doi:10.15252/msb.202010105)
Supplement: Supplementary file 6 — Source Data for Figure 5 [file MSB-17-e10105-s004.zip › Figure5A_sourcedata/GSEA_3071/nosigdb_stateV.GseaPreranked.1622795159954/NB_KI_CLUSTER18.html]

Details for gene set NB\_KI\_CLUSTER18[GSEA]

|  || Dataset | state43071 |
| Phenotype | NoPhenotypeAvailable |
| Upregulated in class | na\_neg |
| GeneSet | NB\_KI\_CLUSTER18 |
| Enrichment Score (ES) | -0.73410314 |
| Normalized Enrichment Score (NES) | -3.7709625 |
| Nominal p-value | 0.0 |
| FDR q-value | 0.0 |
| FWER p-Value | 0.0 |
Table: GSEA Results Summary

  

Fig 1: Enrichment plot: NB\_KI\_CLUSTER18      
 Profile of the Running ES Score & Positions of GeneSet Members on the Rank Ordered List

  

| PROBE | GENE SYMBOL | GENE\_TITLE | RANK IN GENE LIST | RANK METRIC SCORE | RUNNING ES | CORE ENRICHMENT || 1 | LHFPL6 |  |  | 118 | 1.331 | -0.0068 | No |
| 2 | LIMA1 |  |  | 264 | 1.098 | -0.0216 | No |
| 3 | RGS16 |  |  | 348 | 1.018 | -0.0254 | No |
| 4 | CDKN1A |  |  | 363 | 0.993 | -0.0163 | No |
| 5 | A2M |  |  | 532 | 0.846 | -0.0385 | No |
| 6 | COL1A2 |  |  | 1028 | 0.614 | -0.1259 | No |
| 7 | MFGE8 |  |  | 1168 | 0.572 | -0.1458 | No |
| 8 | FCGRT |  |  | 1328 | 0.526 | -0.1700 | No |
| 9 | C1R |  |  | 1696 | 0.437 | -0.2350 | No |
| 10 | FOS |  |  | 2529 | 0.319 | -0.3904 | No |
| 11 | TIMP3 |  |  | 2826 | 0.286 | -0.4437 | No |
| 12 | STOM |  |  | 3230 | -0.256 | -0.5178 | No |
| 13 | COL18A1 |  |  | 3251 | -0.259 | -0.5185 | No |
| 14 | TPM4 |  |  | 3591 | -0.314 | -0.5797 | No |
| 15 | ITGB1 |  |  | 3614 | -0.317 | -0.5801 | No |
| 16 | GSN |  |  | 3695 | -0.331 | -0.5915 | No |
| 17 | VIM |  |  | 3830 | -0.354 | -0.6130 | No |
| 18 | YBX3 |  |  | 3863 | -0.361 | -0.6148 | No |
| 19 | JUNB |  |  | 4027 | -0.394 | -0.6414 | No |
| 20 | SOCS3 |  |  | 4191 | -0.435 | -0.6674 | No |
| 21 | CD99 |  |  | 4339 | -0.484 | -0.6898 | No |
| 22 | MEF2C |  |  | 4351 | -0.487 | -0.6861 | No |
| 23 | CYR61 |  |  | 4384 | -0.497 | -0.6864 | No |
| 24 | IFITM3 |  |  | 4424 | -0.509 | -0.6878 | No |
| 25 | UACA |  |  | 4621 | -0.604 | -0.7182 | No |
| 26 | IGFBP7 |  |  | 4622 | -0.604 | -0.7110 | No |
| 27 | ITGA1 |  |  | 4706 | -0.647 | -0.7193 | No |
| 28 | LGALS1 |  |  | 4784 | -0.699 | -0.7257 | Yes |
| 29 | MT2A |  |  | 4799 | -0.714 | -0.7199 | Yes |
| 30 | THY1 |  |  | 4874 | -0.781 | -0.7249 | Yes |
| 31 | NR2F2 |  |  | 4917 | -0.830 | -0.7231 | Yes |
| 32 | ZFP36L1 |  |  | 4929 | -0.843 | -0.7152 | Yes |
| 33 | NOTCH3 |  |  | 4936 | -0.852 | -0.7063 | Yes |
| 34 | CST3 |  |  | 4951 | -0.868 | -0.6987 | Yes |
| 35 | CALD1 |  |  | 4960 | -0.877 | -0.6898 | Yes |
| 36 | IGFBP5 |  |  | 4978 | -0.897 | -0.6825 | Yes |
| 37 | RRBP1 |  |  | 4981 | -0.898 | -0.6722 | Yes |
| 38 | CYTOR |  |  | 5022 | -0.978 | -0.6683 | Yes |
| 39 | LGALS3 |  |  | 5039 | -1.017 | -0.6593 | Yes |
| 40 | SERPINH1 |  |  | 5059 | -1.050 | -0.6505 | Yes |
| 41 | CEBPB |  |  | 5076 | -1.088 | -0.6407 | Yes |
| 42 | MYH9 |  |  | 5090 | -1.125 | -0.6299 | Yes |
| 43 | FILIP1L |  |  | 5103 | -1.158 | -0.6185 | Yes |
| 44 | PDLIM1 |  |  | 5114 | -1.191 | -0.6063 | Yes |
| 45 | CAVIN1 |  |  | 5116 | -1.195 | -0.5923 | Yes |
| 46 | NUPR1 |  |  | 5123 | -1.217 | -0.5791 | Yes |
| 47 | AXL |  |  | 5132 | -1.249 | -0.5658 | Yes |
| 48 | TIMP1 |  |  | 5134 | -1.251 | -0.5512 | Yes |
| 49 | TGFBI |  |  | 5136 | -1.259 | -0.5365 | Yes |
| 50 | LMNA |  |  | 5141 | -1.277 | -0.5222 | Yes |
| 51 | GADD45B |  |  | 5155 | -1.335 | -0.5089 | Yes |
| 52 | SERPINF1 |  |  | 5158 | -1.351 | -0.4932 | Yes |
| 53 | PRSS23 |  |  | 5189 | -1.489 | -0.4814 | Yes |
| 54 | COL4A2 |  |  | 5198 | -1.545 | -0.4646 | Yes |
| 55 | TFPI |  |  | 5202 | -1.557 | -0.4467 | Yes |
| 56 | BGN |  |  | 5216 | -1.654 | -0.4297 | Yes |
| 57 | IGFBP4 |  |  | 5226 | -1.725 | -0.4110 | Yes |
| 58 | TPM2 |  |  | 5247 | -1.967 | -0.3915 | Yes |
| 59 | MGP |  |  | 5252 | -2.037 | -0.3682 | Yes |
| 60 | PDGFRB |  |  | 5253 | -2.045 | -0.3440 | Yes |
| 61 | COL6A2 |  |  | 5265 | -2.276 | -0.3191 | Yes |
| 62 | COL4A1 |  |  | 5266 | -2.279 | -0.2922 | Yes |
| 63 | CAV1 |  |  | 5279 | -2.605 | -0.2636 | Yes |
| 64 | MYL9 |  |  | 5280 | -2.643 | -0.2324 | Yes |
| 65 | TAGLN |  |  | 5281 | -2.669 | -0.2008 | Yes |
| 66 | COL6A1 |  |  | 5283 | -2.747 | -0.1684 | Yes |
| 67 | FN1 |  |  | 5287 | -2.866 | -0.1351 | Yes |
| 68 | COL3A1 |  |  | 5292 | -3.630 | -0.0929 | Yes |
| 69 | COL6A3 |  |  | 5293 | -3.722 | -0.0488 | Yes |
| 70 | COL1A1 |  |  | 5294 | -4.144 | 0.0002 | Yes |
Table: GSEA details [plain text format]

  

Fig 2: NB\_KI\_CLUSTER18: Random ES distribution      
 Gene set null distribution of ES for **NB\_KI\_CLUSTER18**
